# Novel Focus of Sin Nombre Virus in *Peromyscus eremicus* Mice, Death Valley National Park, California, USA

**DOI:** 10.3201/eid2406.180089

**Published:** 2018-06

**Authors:** Joseph E. Burns, Marco E. Metzger, Sharon Messenger, Curtis L. Fritz, Inger-Marie E. Vilcins, Barryett Enge, Lawrence R. Bronson, Vicki L. Kramer, Renjie Hu

**Affiliations:** California Department of Public Health, Ontario, Richmond, Sacramento, and Redding, California, USA

**Keywords:** Sin Nombre virus, hantavirus, *Peromyscus* *eremicus*, Cricetidae, cactus mouse, Death Valley, Scotty’s Castle, California, viruses, zoonoses, national park, Death Valley National Park

## Abstract

The deer mouse (*Peromyscus* *maniculatus*) is the primary reservoir for Sin Nombre virus (SNV) in the western United States. Rodent surveillance for hantavirus in Death Valley National Park, California, USA, revealed cactus mice (*P. eremicus*) as a possible focal reservoir for SNV in this location. We identified SNV antibodies in 40% of cactus mice sampled.

Hantaviruses constitute a worldwide group of predominantly rodentborne zoonotic pathogens, some of which have emerged as distinctive human health hazards. In North America, Sin Nombre virus (SNV) is the most widespread hantavirus and is of primary public health importance because of the high case-fatality rate (>35%) associated with hantavirus pulmonary syndrome (HPS) (*1*). The principal reservoir of SNV is the deer mouse, *Peromyscus maniculatus* (*2*), a habitat generalist. Evidence of virus infection can be detected in populations of these mice throughout their range (*3*). Several other hantavirus strains have been identified in other species of mice in the family Cricetidae, but pathogenicity of these strains to humans remains unresolved (*4**,**5*).

Field and laboratory studies in North America have confirmed a close association between rodent species and specific hantavirus strains; limited sustained interspecies infection have been documented (*6*). In California, detection of elevated serum antibody titers to hantavirus in rodents other than deer mice have been assumed to represent El Moro Canyon virus in western harvest mice (*Reithrodontomys megalotis*); Isla Vista virus in California voles (*Microtus californicus*); Limestone Canyon virus in brush mice (*P. boylii*); or “spillover” of SNV infection from *P. maniculatus* to other species (*1*). It is not uncommon to detect serum antibodies to SNV in a small percentage (<5%) of sampled species that share habitat with *P. maniculatus* mice, including the closely related *P. boylii, P. fraterculus*, *P. eremicus*, and *P. truei* mice, as well as wood rats, *Neotoma* spp. (*3*). In California, SNV-seropositive rodents typically have not been found to exceed the average *P. maniculatus* infection prevalence of ≈14%, although infection prevalence estimates have slightly exceeded 14% among certain populations of *R. megalotis* mice (*3*,*7*). These presumptive spillover infections are believed to be incidental and not likely to result in sustained transmission in the secondarily infected species (*1*,*3*).

We conducted a survey in Death Valley National Park, California, USA, to document the presence and estimate the infection prevalence of hantavirus in rodents living in and around buildings within select developed areas of the park. The ultimate objective was to assess potential occupational risk to staff and incidental risk to visitors in a highly visited geographic area of the state that was previously unstudied (*8*,*9*).

## The Study 

Death Valley National Park is the largest national park in the contiguous 48 United States and is well known for having some of the hottest desert valleys in North America. Nearly 1 million persons visit the park each year; most visitation occurs during late autumn through mid-spring. Scotty’s Castle, located in the northeastern region of the park (37.031°N, 117.340°W, elevation 950 m), is a popular tourist attraction that has ≈100,000 visitors annually (US National Park Service, pers. comm., 2015). The attraction consists of an extensive 2-story historic villa and associated outbuildings, used as offices, residences, a visitor’s center, and storage, designed in Mission and Spanish Colonial Revival architecture. Below the villa lies a complex of service tunnels that are also included in tours. A year-round natural spring provides surface water flow for ≈0.3 km, creating an oasis in the desert.

We sampled rodents on the Scotty’s Castle grounds in March 2010, April 2011, and October 2011. During each sampling event, we placed 100 aluminum Sherman live-traps (HB Sherman Traps Inc., Tallahassee, FL, USA) throughout the compound, including the riparian zone, inside and around occupied and unoccupied buildings, and in the service tunnels below the villa. We baited traps with dry oats and peanuts, set late in the afternoon, and retrieved captured rodents the following morning. We anesthetized the rodents, then collected reproductive and morphometric data, and identified the rodents to species. We collected a minimum of 11 µL of blood from the retrobulbar sinus of each mouse for ELISA testing for SNV IgG (*10*); all *Peromyscus* spp. mice were humanely euthanized for SNV molecular testing. Blood samples and carcasses were analyzed at the California Department of Public Health Viral and Rickettsial Disease Laboratory (Richmond, CA, USA).

A total of 109 mice were captured during the 3 sampling events (300 trap nights): 100 (91.7%) *P. eremicus*, 5 (4.6%) *P. maniculatus,* and 4 (3.7%) *P. crinitus*. For *P. eremicus* mice, antibodies reactive to SNV antigen, PCR positive tissue, or both were detected in 13 (40.6%) of 32 collected in March 2010, 13 (32.5%) of 40 collected in April 2011, and 20 (71.4%) of 28 collected in October 2011. For *P. maniculatus* mice, antibodies reactive to SNV antigen, PCR positive tissue, or both were detected in 1 (100%) of 1 collected in March 2010, 0 (0%) of 1 collected in April 2011, and 2 (66.6%) of 3 collected in October 2011 (Table). None of the 4 *P. crinitus* mice tested positive for SNV antibodies or viral RNA. We compared viral RNA sequence (Gn) results for 48 *P. eremicus* and 1 *P. maniculatus* mice collected in each of the 3 collection periods to related hantaviruses and found a close consensus (>98%) to Convict Creek viruses 74 and 107 ([GenBank accession nos. L33474 and L33684) (*11*) (Figure).

**Table:** Sin Nombre virus test results among *Peromyscus* mouse species, by test type, for sampling conducted in March 2010, April and October 2011, Death Valley National Park, California, USA

Species	No. positive/no. tested (%)								
	March 2010				April 2011*		October 2011		
Seropositive	RNA positive	Both	Seropositive	Seropositive	RNA positive	Both
*P. eremicus*	2/32 (6.2)	0/32 (0)	11/32 (34.4)		13/40 (32.5)		5/28 (17.9)	6/28 (21.4)	9/28 (32.1)
*P. maniculatus*	0/1 (0)	0/1 (0)	1/1 (100)		0/1 (0)		1/3 (33.3)	1/3 (33.3)	0/3 (0)
*P. crinitus*	0/0	0/0	0/0		0/2 (0%)		0/2 (0%)	0/2 (0%)	0/2 (0%)

*PCR testing was not performed on April 2011 samples.

**Figure:**
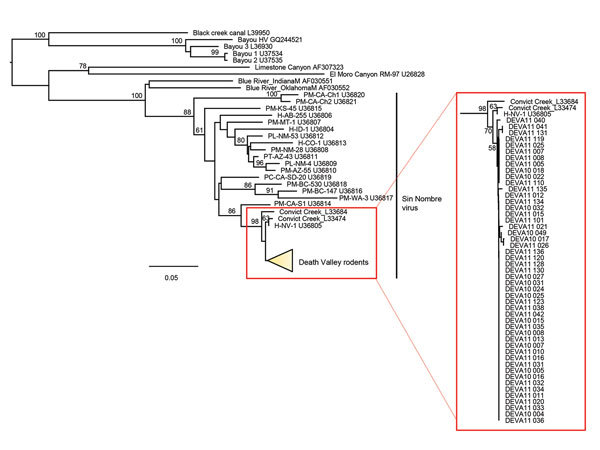
Phylogenetic tree of Gn glycoprotein sequences comparing hantaviruses sampled from 48 *Peromyscus eremicus* and 1 *P. maniculatus* (DEVA 10 022) mice collected in Death Valley National Park, California, USA (detail in inset box; GenBank accession nos. MG992890–MG992938). Representative reference sequences of hantaviruses in the United States were downloaded from GenBank (accession numbers included in taxon labels). The tree was reconstructed by analysis of 370 bases of the glycoprotein precursor (GPC) gene by using the neighbor-joining method, employing the HKY model, to estimate genetic distances. We estimated support for relationships by using a nonparametric bootstrap analysis (1,000 replicates). Nodes with bootstrap percentages >50% are indicated. Similar tree topologies were generated from maximum-likelihood (RAxML) and Bayesian (Mr. Bayes) phylogenetic analyses (not shown), implemented by using Geneious version 10.0 (Biomatters; Newark, New Jersey, USA). Scale bar represents genetic distance (substitutions per site). DEVA, Death Valley National Park.

## Discussion

The *P. maniculatus* deer mouse is recognized as the primary reservoir for SNV in the western United States. Published estimates for SNV seroprevalence are consistently higher in *P. maniculatus* mice than for any other *Peromyscus* species. Rodent hantavirus surveillance in California during 2001–2010 (*7*) detected the serum antibody to SNV among 14% (1,058/7,621) of deer mice statewide; concurrently, SNV seroprevalence for *P. eremicus* mice was significantly lower at 3.7% (102/2,723) and did not exceed this highest site-specific estimate at any individual surveillance site. 

We identified serum antibodies to SNV in 40% of *P. eremicus* mice sampled. The consistently high seroprevalence over 3 sample periods suggests that SNV is efficiently transmitted and maintained within this population. The sequence characterization of viral RNA from seropositive *P. eremicus* mice further substantiates that the virus closely resembles type strains of pathogenic SNV associated with HPS.

The factors necessary to sustain a virus–reservoir relationship are both intrinsic and extrinsic. Hantaviruses are believed to have coevolved with their respective rodent hosts (*1*). *P. eremicus* mice are most closely phylogenetically related to *P. maniculatus* and *P. leucopus* mice, reservoirs for hantaviruses SNV and Monogahela virus, respectively, which are recognized to cause HPS (*12*). The close genetic similarity among these species may best enable *P. eremicus* mice among *Peromyscus* spp. mice in California to serve as a viable alternative host for SNV or to harbor a coevolved hantavirus of similar SNV lineage.

Hantaviruses are transmitted between rodent hosts through direct contact. Thus, a minimum population density is required to sustain transmission within an isolated group. The optimal habitat provided by the oasis setting of our study enables the typically solitary cactus mouse (*13*) to achieve a greater population density not generally found in most of the sylvan desert habitats where these mice are native (0.21–3.3/hectare) (*14*). Further studies in similar high density/optimal peridomestic habitats, which are associated with higher SNV infection prevalence (*1*), are needed to establish whether this high level of infection is reflective of the unique environment or an alternate phenotypic expression or strain, considering the enhanced transmission and maintenance of SNV in cactus mice detected at this location. 

These findings underscore the importance of rodent exclusion and management in and around rural and semirural buildings where risk for contact between rodents and humans is high, even in the absence of *P. maniculatus* mice (*9**,**15*). Park leadership and staff were notified of our study results and given training on hantavirus awareness and prevention, and hantavirus pamphlets were made available for visitors to the park.
